# Interdependent and independent roles of type I interferons and IL-6 in innate immune, neuroinflammatory and sickness behaviour responses to systemic poly I:C

**DOI:** 10.1016/j.bbi.2015.04.009

**Published:** 2015-08

**Authors:** Carol Murray, Éadaoin W. Griffin, Elaine O’Loughlin, Aoife Lyons, Eoin Sherwin, Suaad Ahmed, Nigel J Stevenson, Andrew Harkin, Colm Cunningham

**Affiliations:** a School of Biochemistry and Immunology, Trinity College Dublin, Ireland; b Trinity College Institute of Neuroscience, Trinity College Dublin, Ireland

**Keywords:** Interferon, Viral, IFN-β, IFN-α, IL-6, Sickness, Behaviour, Hypoactivity, Depression, Cytokine, Neuroinflammation, Hippocampus, IDO, Kynurenine, STAT1, Burrowing

## Abstract

Type I interferons (IFN-I) are expressed in the brain during many inflammatory and neurodegenerative conditions and have multiple effects on CNS function. IFN-I is readily induced in the brain by systemic administration of the viral mimetic, poly I:C (synthetic double-stranded RNA). We hypothesised that IFN-I contributes to systemically administered poly I:C-induced sickness behaviour, metabolic and neuroinflammatory changes. IFN-I receptor 1 deficient mice (IFNAR1^−/−^) displayed significantly attenuated poly I:C-induced hypothermia, hypoactivity and weight loss compared to WT C57BL/6 mice. This amelioration of sickness was associated with equivalent IL-1β and TNF-α responses but much reduced IL-6 responses in plasma, hypothalamus and hippocampus of IFNAR1^−/−^ mice. IFN-β injection induced trivial IL-6 production and limited behavioural change and the poly I:C-induced IFN-β response did not preceed, and would not appear to mediate, IL-6 induction. Rather, IFNAR1^−/−^ mice lack basal IFN-I activity, have lower STAT1 levels and show significantly lower levels of several inflammatory transcripts, including stat1. Basal IFN-I activity appears to play a facilitatory role in the full expression of the IL-6 response and activation of the tryptophan-kynurenine metabolism pathway. The deficient IL-6 response in IFNAR1^−/−^ mice partially explains the observed incomplete sickness behaviour response. Reconstitution of circulating IL-6 revealed that the role of IFNAR in burrowing activity is mediated via IL-6, while IFN-I and IL-6 have additive effects on hypoactivity, but the role of IFN-I in anorexia is independent of IL-6. Hence, we have demonstrated both interdependent and independent roles for IFN-I and IL-6 in systemic inflammation-induced changes in brain function.

## Highlights

• IFNAR1^−/−^ mice show diminished sickness behaviour response to poly I:C.
• Poly I:C fails to induce a robust IL-6 response in IFNAR1^−/−^ mice.
• Basal STAT1 levels are lower in IFNAR1^−/−^ mice and several inflammatory transcripts are also lower at baseline in IFNAR1^−/−^.
• Adding IL-6 with poly I:C reconstitutes more robust sickness in IFNAR1^−/−^.

## Graphical abstract

**Figure:**
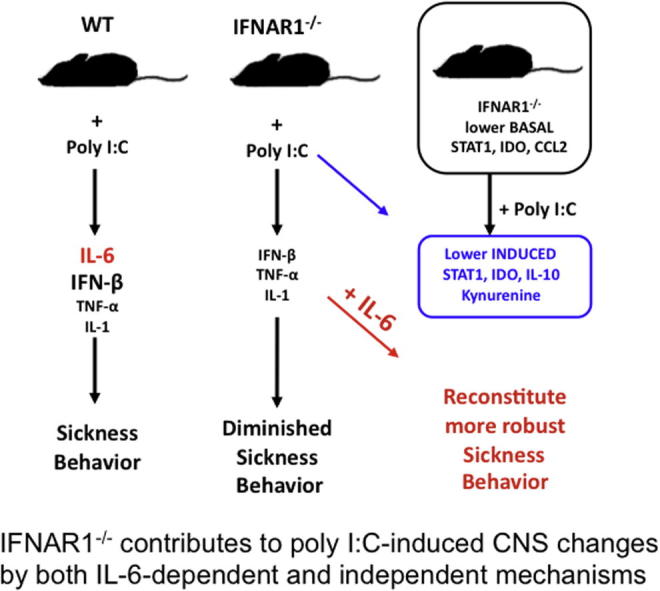

## 1 Introduction

Type one interferons, IFN-α and IFN-β (IFN-I) are produced by most cell types in response to viral and bacterial pathogens (Colonna et al., 2002) and have multiple roles in antiviral defense, cell growth regulation and immune activation. IFN-I signals through the heterodimeric receptor composed of IFNAR1 and IFNAR2, inducing cross phosphorylation of Tyk2 and Jak1, which leads to tyrosine phosphorylation and activation of signal transducers and activators of transcription (STATs) STAT1 and STAT2. These, in turn, form transcriptional activator complexes through a series of signalling cascades leading to the activation of transcription factors, translocation to the nucleus and binding of the interferon-stimulated response element (ISRE), to induce transcription of >500 IFN stimulated genes (ISGs) (Taniguchi et al., 2001).

It has emerged that IFN-I and downstream genes are readily induced in the brain (Cunningham et al., 2007; Wang et al., 2008) and have multiple effects on brain function and pathology (Owens et al., 2014). While recombinant IFN-β is a first line treatment for relapsing-remitting Multiple Sclerosis, IFN-α, when used to treat cancer and hepatitis, has been shown to induce sickness behaviour, depression, anxiety, cognitive impairment and even delirium (Capuron and Miller, 2004; Schaefer et al., 2002; Raison et al., 2005). Chronic overexpression of IFN-α produces CNS pathology (Campbell et al., 1999) and intracerebroventricular administration of blocking antibodies against IFNAR protected against age-associated impairment of neurogenesis and cognitive deficits (Baruch et al., 2014). Acute changes in IFN-I also impact on temperature, sleep (Krueger et al., 1988), circadian rhythm (Koyanagi and Ohdo, 2002) and feeding behaviour (Plata-Salaman, 1992). IFN-β has been shown to increase interictal-like spontaneous activity in hippocampal neurons (Costello and Lynch, 2013) and to downregulate the brain-derived neurotrophic factor (BDNF) receptor, TrkB (Dedoni et al., 2012).

Poly I:C is a synthetic double stranded RNA, commonly used as a viral mimetic, that is recognised by TLR3 receptors on endosomal membranes and by the cytoplasmic proteins RIG I and MDA5 (Alexopoulou et al., 2001; Yoneyama et al., 2004; Andrejeva et al., 2004; Kato et al., 2005). Systemically administered poly I:C, induces, in mice and rats, the classical features of sickness behaviour (Hart, 1988), including fever/hypothermia, malaise, hypoactivity, anhedonia, sleep/wake cycle disruption and anorexia (Fortier et al., 2004; Cunningham et al., 2007; Majde, 2000; Traynor et al., 2006). There is also evidence for effects of systemic (Kranjac et al., 2012; Dilger and Johnson, 2010) or intracerebroventricular administration (Okun et al., 2010) of poly I:C on cognitive function. The poly I:C-induced fever in rats is partially dependent on IL-1β (Fortier et al., 2004) and temperature and sleep/wake regulation have been described to be altered in IFNAR1^−/−^ mice (Traynor et al., 2006), but there has been little investigation of the role of IFN-I in poly I:C-induced sickness behaviour and neuroinflammatory changes. Here, we investigated the role of IFN-I in the CNS effects of systemic poly I:C using IFNAR1^−/−^ mice and C57BL6 controls. We show a role of IFNAR1 in hypoactivity, anhedonia, hypothermia and anorexia upon systemic challenge with this double stranded RNA viral mimetic. We also observed significant impacts of IFNAR deficiency on IL-6 expression. Understanding the interactions between these cytokines in mediating the CNS effects of viral infection may be important in mitigating the discomforting effects of acute illness and may provide insights into the acceleration of chronic neurodegenerative disease by the stimulation of acute anti-viral responses (Field et al., 2010).

## 2 Methods

All animal procedures were performed under licence from the Irish Department of Health & Children after a full ethical review by the TCD animal research ethics committee, in compliance with the Cruelty to Animals Act, 1876 and the European Community Directive, 86/609/EEC. Female C57BL/6 mice (Harlan, Bicester, UK) were housed in groups of five, with food and water *ad libitum*. Female mice were used to minimise aggression. Animals were kept at 21 °C with 12:12 h light-dark cycle. IFNAR1^−/−^ mice were obtained from Prof Paul J Hertzog of Monash Medical Centre, Monash University, Clayton, Victoria, Australia. Generation of mutant mice was as previously described (Hwang et al., 1995): 129Sv ES cells were transferred into the Balb/C background and offspring backcrossed onto a C57BL6/J background for >7 generations.

### 2.1 Systemic inflammatory challenges

#### 2.1.1 Poly I:C

Poly I:C obtained from Amersham Biosciences (Little Chalfont, Buckinghamshire, UK) was prepared for injection by re-suspension in sterile saline, and was heated to 50 °C at a concentration of 2 mg/ml and was allowed to cool to room temperature to ensure proper annealing of double-stranded RNA. Poly I:C was stored at −20 °C until use. Experimental groups were challenged intraperitoneally (i.p.) with either poly I:C (12 mg/kg) or sterile saline, based on our prior observations with this dose (Cunningham et al., 2007). Blood and hypothalamic and hippocampal tissues were collected, from a separate group of animals, 3 h post-poly I:C challenge for analysis of inflammatory markers, and also at 1 h in a subset of the study.

#### 2.1.2 Cytokines

Experimental groups were injected with recombinant mouse IL-6 i.p (Peprotech, Rocky Hill, NJ, USA) at a dose of 50 μg/kg prepared in 0.9% sterile saline, judged to be a mild dose based on our own prior observations (Skelly et al., 2013 and references therein). Control animals were injected with sterile saline (Sigma, Poole, UK) 10 ml/kg body weight. Additional animals were injected with recombinant mouse IFN-β i.p (PBL Interferon source, NJ, USA) at a dose of 25,000 units or with vehicle solution, phosphate buffered saline containing 0.037% Glycerol and 0.0094% BSA, 10 ml/kg body weight.

### 2.2 Behavioural & sickness analyses

#### 2.2.1 Burrowing

Black plastic cylinders, 20 cm long, 6.8 cm diameter, sealed at one end, were filled with 300 g of normal diet food pellets, and placed in individual mouse cages. The open end was raised by 3 cm above the floor preventing non-purposeful displacement of the contents. Mice were placed individually in the cages at appropriate times post-poly I:C, IFN-β or IL-6 challenges. The food remaining in the cylinders after 2 h was weighed and the amount displaced (burrowed) was calculated. Baseline tests were run 24 h before inflammatory challenges. Burrowing is typically performed during or just before the dark phase and is thus generally performed between 6 and 12 h post-poly I:C or cytokine challenge.

#### 2.2.2 Open field

To investigate locomotor and rearing activity the open field task was used. Briefly, mice were placed in a box (58 cm × 33 cm × 19 cm) 3 h post-poly I:C or IL-6 challenges and 9 h post IFN-β. The former times were chosen on the basis of observed suppression of open field activity in prior poly I:C studies (Cunningham et al., 2007) while the latter was chosen after observing no suppression at early time points but some suppression of activity during the peak dark phase activity in pilot IFN-β experiments. Rearing and square crossings (distance travelled) were recorded for 3 min. Baseline tests were run 24 h before challenges.

#### 2.2.3 Temperature

Body temperature was measured using a rectal probe (RET-3-ISO Rectal Probe Physitemp, NJ) on three occasions in the week prior to poly I:C injections to habituate mice to this procedure. Mice were then assessed at the time of injection and at intervals afterwards. These intervals were 3, 6 and 9 h post-poly I:C, based on prior experiments (Cunningham et al., 2007) and were not changed significantly (4, 9, and 24 h) post-IFN-β challenges.

#### 2.2.4 Body weight

**Fig. 1:**
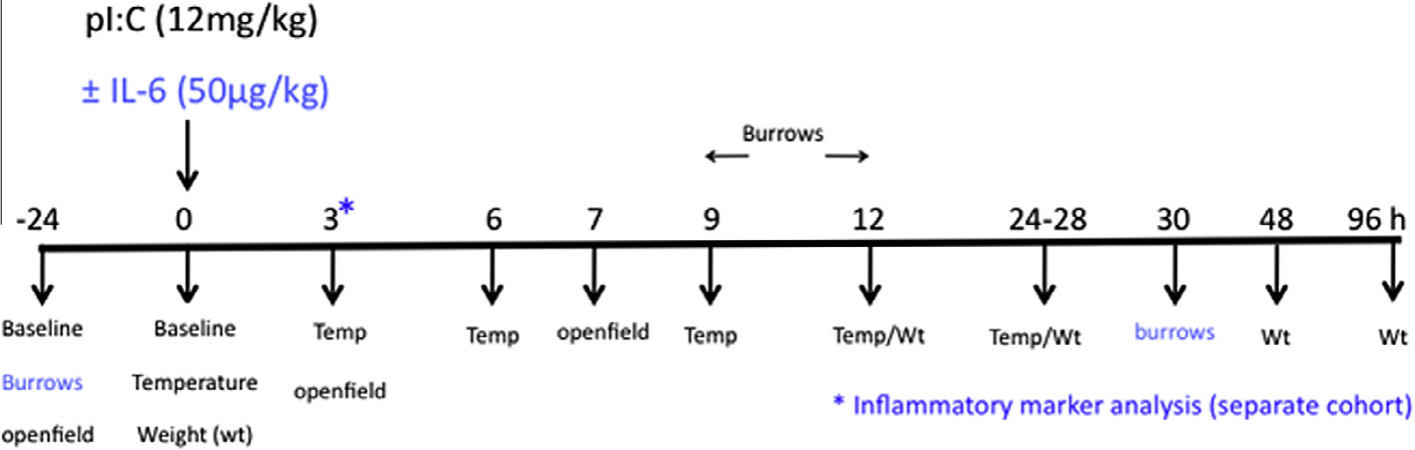
Experimental timeline. Schematic showing the timeline of treatments and parameters measured during the poly I:C treatment experiments conducted in the current studies. Burrows were analysed only in the ±IL-6 experiment and are shown in blue. Tissue for inflammatory mediator analysis (*) was collected from a separate cohort. (For interpretation of the references to colour in this figure legend, the reader is referred to the web version of this article.)

Body weight was recorded at the time of injection and at the following intervals afterwards; 12, 28 and 48 h post-poly I:C and IL-6 challenges. For the IFN-β challenges body weight was measured at 7 and 24 h post -challenge. A timeline for the assays performed during poly I:C challenges is shown in Fig. 1.

### 2.3 RNA extraction and quantitative PCR

Mice were terminally anaesthetised (i.e administered a fatal dose of anaesthetic) using Euthatal and transcardially perfused with heparinised saline 3 h post-challenge with poly I:C or IFN-β. The hypothalami and hippocampi were collected and stored at −80 °C. RNA was extracted from these samples using Qiagen RNeasy® Plus mini kits (Qiagen, Crawley, UK) and yields were determined by spectrophotometry at 260 nm as previously described (Murray et al., 2011). cDNA synthesis was performed using a High Capacity cDNA Reverse Transcription Kit (Applied Biosystems, Warrington). 200 ng of total RNA was reverse transcribed in a 10 μl reaction volume. One microlitre of the RT reaction (equivalent to 20 ng of RNA) was used for PCR. Quantification was carried out as previously described (Cunningham et al., 2005) after normalisation to GAPDH expression.

### 2.4 ELISA

IFNAR1^−/−^ and WT animals were terminally anaesthetised 3 h post-poly I:C (12 mg/kg i.p.), IFN-β (25,000 units, i.p.) or saline challenges and the thoracic cavity opened and blood was collected into heparinised tubes directly from the right atrium of the heart. Blood was centrifuged to remove cells and the remaining plasma was aliquoted and stored at −20 °C. Samples from IFNAR1^−/−^ and WT animals were analysed for plasma IL-6 and TNF-α (R&D Systems U.K) and IFN-β (PBL Biomedical Laboratories, USA) using commercially available enzyme-linked immunosorbent assay kits, as per manufacturer guidelines.

### 2.5 Western blotting

Hippocampal punches, weighing 20–30 mg were homogenised in 100 μl of lysis buffer (50 mM Tris–HCl, 150 mM NaCl, 1% Triton ×10) with protease and phosphatase inhibitors. The homogenate was then centrifuged at 4 °C for 10 min at 10,000 rpm. Total protein content was quantified using a DC protein Assay (Biorad) and samples were boiled in loading buffer containing β-mercaptoethanol. 50 μg of each lysate was loaded onto a 10% SDS–PAGE gel and the proteins were transferred to a PVDF membrane following separation by electrophoresis. Membranes were blocked in 5% non-fat milk in TBS and 0.1% Tween-20 for 1 h at room temperature. The membranes were then incubated overnight at 4 °C with primary antibodies STAT1 (SC346, Santa Cruz), phosphorylated STAT1 (#9167 cell signalling) at 1/1000 dilution and Beta Actin (A5441 Sigma) at 1/5000 dilution. Membranes were then washed in TBS-T and incubated with secondary antibodies: goat anti-rabbit (P0448 Dako, 1/1000 dilution) for both STAT1 and phosphorylated STAT1 and goat anti-mouse (Jackson, 1/10,000 dilution) for Beta Actin. Membranes were then developed using Supersignal West Dura Extended Duration ECL (Pierce) and quantified using Image J software (imagej.nih.gov/ij/).

### 2.6 HPLC

Tryptophan and kynurenine were measured in serum and brain samples using HPLC coupled to UV/fluorescence detection as previously described (Gibney et al., 2014). Samples were diluted in mobile phase containing 50 nM glacial acetic acid, 100 mM zinc acetate (Sigma, Ireland) and 3% acetonitrile dissolved in double-distilled NANOpure water HPLC grade H_2_O (Sigma, Ireland) at pH 4.9. Plasma was diluted 1:1 in mobile phase containing 6% perchloric acid (for protein precipitation) spiked with 200 ng/20 μl of *N*-methyl 5-HT (Sigma, Ireland) as internal standard. Samples were weighed and sonicated in mobile phase containing 6% perchloric acid spiked with 200 ng/20 μl of N-methyl 5-HT as internal standard. Samples were centrifuged at 20,000 rpm for 20 min and the supernatants were placed into new eppendorf tubes, using a syringe fitted with a 0.45 μM filter (Phenomenex, UK). 20 μl of the filtered supernatant was injected using a waters autosampler and a Reverse Phase analytical column (Kinetex™ Core Shell Technology column with specific area of 100 mm × 4.6 mm and particle size of 2.6 μm, Phenomenex, UK) was used for separation of metabolites. A PDA-UV detector (Shimadzu SPD-M10A VP), calibrated to integrate at 230, 250 and 365 nM, as well as a fluorescent detector (Shimadzu RF-20A XS prominence fluorescence detector), set to excitation wavelength 254 nM; emission wavelength 404 nM, were used to detect tryptophan and kynurenine. Chromatographs were generated by CLASS-VP software (Shimadzu, UK). Results are expressed as ng of analyte per ml of plasma and ng of analyte per gram of tissue.

### 2.7 Statistical analysis

#### 2.7.1 Poly I:C challenges

We made *a priori* predictions that poly I:C would produce robust suppression of rearing activity at 3 h and hypothermia at 8 h. Therefore we performed two-way ANOVA with strain and treatment as factors for these analyses. Body weight was analysed by three-way ANOVA with strain and treatment as between subjects factors and time as within subjects factor. All ELISA, RT-PCR, HPLC and Western blots were also analysed by two-way ANOVA or Students *t*-test.

#### 2.7.2 IFN-β challenges

Open field rearing activity and burrowing in saline and IFN-β-treated animals was analysed by Students *t*-test at 9 and 7 h respectively. IL-6 protein expression and mRNA level were analysed at 3 h by Students *t*-test.

#### 2.7.3 IL-6 challenges

Burrowing, rears and body weight responses to acutely administered IL-6 were analysed by three-way ANOVA with strain and treatment as between-subjects factors and time as a repeated measure. For poly I:C ± IL-6 challenges we had *a priori* predictions and performed two-way ANOVA analysis of burrowing at 9 h and rearing at 3 h. Body weight data were analysed using three-way ANOVA with strain, treatment and time as factors.

## 3 Results

### 3.1 Poly I:C-induced type I interferons and sickness behaviour

**Fig. 2:**
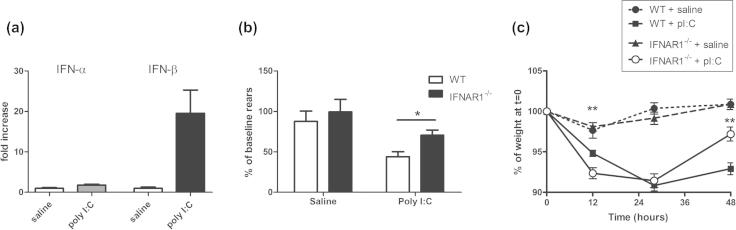
Poly I:C-induced type I interferons and sickness behaviour in WT and IFNAR1^−/−^ mice. (a) The hypothalamic transcription of IFN-α and IFN-β were assessed at 6 h post-poly I:C (12 mg/kg i.p.). (b) The sickness behaviour response of C57BL/6 and IFNAR1^−/−^ mice to poly I:C was assessed (b) open field rearing activity and (c) % body weight loss. Differential effects of poly I:C on (b) rearing were assessed using two-way ANOVA (with strain and treatment as between-subjects factors) and (c) body weight was assessed using 3 way ANOVA analysis with time, treatment and strain as factors in WT and IFNAR1^−/−^ mice. Data are expressed as mean ± SEM, *n* = 5 for WT + saline, *n* = 8 for WT + pI:C, *n* = 13 for IFNAR1^−/−^ + saline and *n* = 14 for IFNAR1^−/−^ + pI:C. Full ANOVA analysis appears in the main text. Statistically significant differences by Bonferroni *post-hoc* are denoted by ^∗∗^*p* < 0.01 and ^∗^*p* < *0.05 w.r.t* WT + pI:C *.*

Poly I:C induced robust hypothalamic expression of IFN-β mRNA but not IFN-α mRNA (Fig. 2a) and induced some features of sickness in all treated animals. IFNAR1^−/−^ mice appeared to show less hunched posture and piloerection than WT animals when challenged with poly I:C. We quantified the sickness behaviour response using a number of objective measures.

Temperature was measured, by rectal probe, at 3, 6, and 9 h post-poly I:C challenge and early measures showed variability and possible stress-related hyperthermia in most animals. Nonetheless, WT poly I:C-treated mice showed significant hypothermia at 9 h and this was not robust in poly I:C-treated IFNAR1^−/−^ mice (Supplementary Fig. 1). Based on prior studies (Cunningham et al., 2007), we made an *a priori* prediction of robust hypothermia at 9 h and two-way ANOVA at 9 h showed a significant effect of treatment (*F* = 13.91, df 1,40, *p* = 0.0006), no effect of strain (*F* = 1.37, df 1,40, *p* = 0.2489) but a significant interaction between treatment and strain (*F* = 8.22, df 1,40, *p* = 0.0066) indicating the impaired hypothermic response in IFNAR1^−/−^ mice.

Rears in the open field were recorded for 3 min, 3 h post-challenge with poly I:C or saline. There was a robust decrease in the number of rears in poly I:C-treated mice but this was less marked with IFNAR1^−/−^ poly I:C-treated mice compared to WT poly I:C-treated (Fig. 2b). Two-way ANOVA revealed a significant effect of strain (*F* = 4.51, df 1,40, *p* = 0.04) and of treatment (*F* = 14.85, df 1,40, *p* = 0.0004) but no significant interaction (*F* = 0.88, df 1,40, *p* = 0.3549). Bonferroni *post-hoc* analysis indicated that suppression of rears by poly I:C was significantly more robust in WT animals than in IFNAR1^−/−^ animals (*p* < 0.05).

All poly I:C-treated mice showed a marked reduction in weight but recovery was significantly better in IFNAR1^−/−^ mice at 48 h (Fig. 2c). Three-way ANOVA with strain, treatment and time as factors revealed no significant effect of strain (*F* = 0.23148, df 1,43, *p* = 0.6331) but a significant effect of treatment (*F* = 118.4, df 1,43, *p* < 0.0001) and of time (*F* = 102.95, df 3,43, *p* < 0.0001) and an interaction of all three factors (*F* = 12.99, df 1,43, *p* < 0.0001). Therefore activity of IFN-I influences the time-course of recovery during poly I:C-induced sickness.

### 3.2 Systemic and CNS cytokine responses

#### 3.2.1 Plasma

**Fig. 3:**
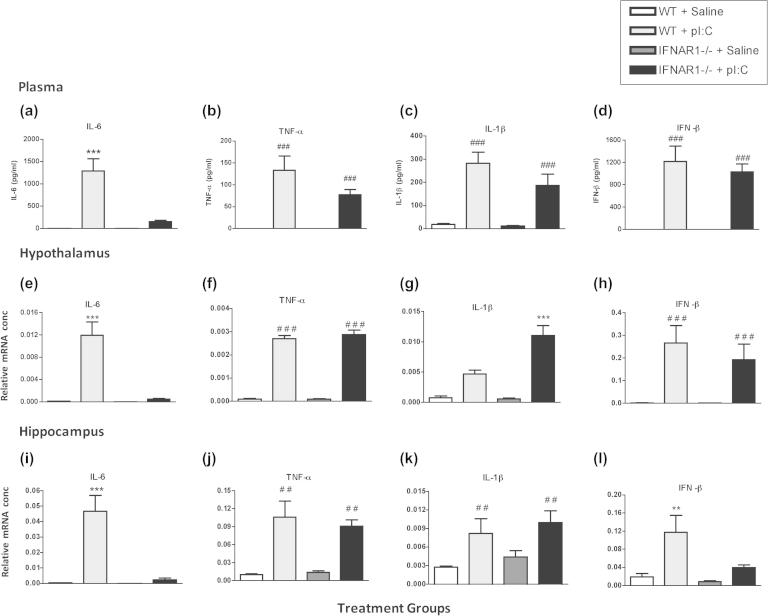
Poly I:C-induced systemic and CNS synthesis of inflammatory cytokines. ELISA analysis of plasma IL-6, TNF-α, IL-1β and IFN-β (a-d) and CNS cytokine transcription 3 h after intraperitoneal challenge with poly I:C (12 mg/kg). Analysis of transcripts is shown for hypothalamus (e–h) and hippocampus (i–l). Data are expressed as mean ± SEM, *n* = 5 for WT and *n* = 6 for IFNAR1^−/−^. Comparisons of pI:C treatment for WT and IFNAR1^−/−^ mice were carried out by two-way ANOVA, with treatment and strain as factors. Main effects of treatment are denoted by ^###^*p* < 0.001, ^##^p < 0.01 and ^#^*p* < 0.05 and interactions between treatment and strain are denoted by ^∗∗∗^*p* < 0.001 and ^∗∗^*p* < 0.001.

Plasma from WT and IFNAR1^−/−^ mice 3 h post-challenge with poly I:C or saline was analysed for systemic levels of pro-inflammatory cytokines (IL-6, TNF-α, IL-1β  and IFN-β) by ELISA (Fig. 3). The plasma concentrations of IL-6, TNF-α, IL-1β  and IFN-β in the saline-treated animals were below detection levels. There was almost complete ablation of the IL-6 response in poly I:C-treated IFNAR1^−/−^ mice. Two-way ANOVA analysis of plasma IL-6 levels revealed a significant effect of treatment (*F* = 28.18, df 1,18, *p* < 0.0001) and of strain (*F* = 21.04, df 1,18, *p* = 0.0002) and an interaction between treatment and strain (*F* = 21.04, df 1,18, *p* = 0.0002). Analysis of plasma TNF-α, IL-1β and IFN-β by two-way ANOVA demonstrated a significant effects of treatment (*F* ⩾ 42.03, df 1,18, *p* < 0.0001) but no effects of strain and no interaction, indicating that poly I:C-induced increases in plasma TNF-α, IL-1β and IFN-β are not affected by IFNAR1.

#### 3.2.2 Brain

Hypothalamic and hippocampal brain regions were also extracted from WT and IFNAR1^−/−^ mice 3 h post-challenge with poly I:C or saline. IFN-β, IL-6, TNF-α and IL-1β expression was measured by RT-PCR. As in the plasma, there was a near- complete ablation of IL-6 in the IFNAR1^−/−^ poly I:C-treated compared to the WT poly I:C-treated in the hypothalamus (Fig. 3e) and in the hippocampus (Fig. 3i). Two-way ANOVA of IL-6 mRNA revealed a significant effect of treatment (*F* = 32.08, df 1,18, *p* < 0.0001), of strain (*F* = 28.18, df 1,18, *p* < 0.0001) and an interaction (*F* = 27.39, df 1,18, *p* < 0.0001) of these two factors in the hypothalamus and this effect was replicated in the hippocampus: treatment (*F* = 26.75, df 1,18, *p* < 0.0001), strain (*F* = 22.46, df 1,18, *p* < 0.0002) and interaction of strain and treatment (*F* = 21.79, df 1,18, *p* < 0.0002).

Conversely, TNF-α was increased by poly I:C in the hypothalamus (Fig. 3f) and the hippocampus (Fig. 3j) in both WT and IFNAR1^−/−^ mice. Two-way ANOVA analysis shows a significant effect of treatment (*F* = 428.72, df 1,18, *p* < 0.0001) but no effect of strain (*F* = 0.46, df 1,18, *p* = 0.5073) and no interaction of strain or treatment (*F* = 0.52, df 1,18, *p* = 0.4782). Similarily in the hippocampus (Fig. 3j), two-way ANOVA shows poly I:C induced increases in TNF-α, with a significant effect of treatment (*F* = 53.39, df 1,17, *p* < 0.0001) but no effect of strain (*F* = 0.24, df 1,17, *p* = 0.6284) and no interaction between strain and treatment (*F* = 0.64, df 1,17, *p* = 0.4362), indicating that poly I:C-induced increases in the expression of TNF-α and IFN-β are not affected by IFNAR1.

IL-1β was also increased by poly I:C in the hypothalamus, but to a greater degree in IFNAR1^−/−^ than WT mice (Fig. 3g). There was a significant effect of treatment (*F* = 57.17, df 1,18, *p* < 0.0001) and of strain (*F* = 10.83, df 1,18, *p* = 0.0041) and an interaction between treatment and strain (*F* = 10.65, df 1,18, *p* = 0.0043). In the hippocampus (Fig. 3k) poly I:C treatment once again had a significant effect on IL-1β expression (*F* = 12.78, df 1,17, *p* < 0.0023) but there was no effect of strain (*F* = 1.24, df 1,17, *p* = 0.2813) and no interaction (*F* = 0.45, df 1,17, *p* = 0.9722) indicating that the poly I:C induced increase in IL-1β mRNA is exacerbated in IFNAR1^−/−^ mice, but only in the hypothalamus.

Poly I:C robustly induced IFN-β in the hypothalamus of both WT and IFNAR1^−/−^ mice (Fig. 3h). There was a significant effect of treatment (*F* = 17.45, df 1,17, *p* < 0.0006) but no effect of strain (*F* = 0.46, df 1,17, *p* = 0.5059) and no interaction (*F* = 0.45, df 1,17, *p* < 0.5136). In the hippocampus, poly I:C induced IFN-β (Fig. 3l) with a main effect of treatment (*F* = 16.89, df 1,16, *p* < 0.0008), strain (*F* = 7.91, df 1,16, *p* < 0.0125) and an interaction of strain × treatment (*F* = 4.58, df 1,16, *p* < 0.0482), indicating a lack of poly I:C-induced IFN-β response in the hippocampus but not the hypothalamus of IFNAR1^−/−^ mice.

These results suggest that IFNAR1 expression appears to be necessary for robust plasma and CNS IL-6 responses to poly I:C and for the full expression of sickness behaviour, but not for IL-1β nor TNF-α responses. This is not the case for IL-6 and sickness behaviour responses to LPS in IFNAR1^−/−^ mice: IL-6 levels and locomotor activity are similarly altered by LPS in WT and IFNAR1^−/−^ mice (Supplementary Fig. 2). There is evidence for an exaggerated hypothalamic IL-1β response to poly I:C in IFNAR1^−/−^ mice.

### 3.3 Does acutely induced IFN-β induce IL-6 and sickness behaviour?

**Fig. 4:**
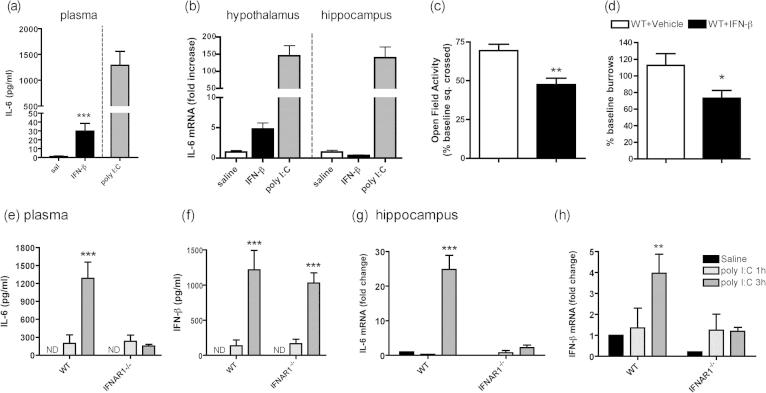
The role of acutely increased IFN-β in induction of IL-6 and sickness behaviour. Plasma IL-6 (a) and IL-6 expression in the hypothalamus and hippocampus (b) of C57BL/6 mice 3 h post-IFN-β challenge (25,000 units, i.p), compared to that induced by poly I:C (12 mg/kg). Data are expressed as mean ± SEM and were analysed by unpaired Student’s *t* test, *n* = 4 for WT + vehicle and *n* = 5 for WT + IFN-β. The sickness behaviour response of C57BL/6 mice to IFN-β (25,000 units, i.p.) on (c) open field activity (expressed as % of baseline squares crossed at 9 h) and (d) burrowing at 7 h. Data are expressed as mean ± SEM and were analysed by Students *t*-test, *n* = 17 for WT + vehicle, *n* = 15 for WT + IFN-β. IL-6 and IFN-β levels in the plasma (e, f) and expression in the hippocampus (g, h) 1 and 3 h post-poly I:C challenge in WT and IFNAR1^−/−^ mice. Data are expressed as mean ± SEM and were analysed by two-way ANOVA with strain and treatment as factors, *n* = 5 for WT + saline, *n* = 4 for WT + pI:C 1 h, *n* = 5 for WT + pI:C 3 h and *n* = 4 for IFNAR1^−/−^ + saline, *n* = 4 IFNAR1^−/−^ + pI:C 1 h and *n* = 5 for IFNAR1^−/−^ + pI:C 3 h. Bonferroni *post-hoc* are denoted by ^*∗∗∗*^*p* < 0.001 and ^∗∗^*p* < 0.001.

The milder sickness behaviour and loss of poly I:C-induced IL-6 in IFNAR1^−/−^ animals suggested a role for IFN-I in IL-6 expression. Therefore we examined whether systemically administered IFN-β could induce IL-6 in wild-type C57BL6 mice. IFN-β increased plasma IL-6 (Fig. 4a; *p* < 0.05 by unpaired Student’s *t* test) and elevated expression of IL-6 mRNA in the hypothalamus compared to vehicle-injected controls (Fig. 4b; *p* < 0.05 by unpaired Student’s *t* test). However, this increase in IL-6 was not replicated in the hippocampus and levels of plasma IL-6 and brain IL-6 mRNA were trivial compared to those induced by poly I:C (Fig. 4a and b).

A number of measures of sickness behaviour were assessed. There were no effects of IFN-β (25,000 units) on body temperature or body weight (data not shown). Open field activity was recorded for 3 min, 9 h post-challenge with IFN-β or vehicle (Fig. 4c) after pilot experiments indicated mild effects on activity only in the dark phase. There was a slight decrease in activity in the IFN-β-treated mice with respect to controls, *p* < 0.0011 by unpaired Student’s *t* test. Burrowing activity was measured at baseline and we made an *a priori* prediction of impairment at 7 h post-challenge with IFN-β (25,000 units; Fig. 4d). Students *t*-test at 7 h showed a small but statistically significant effect of treatment (*p* = 0.0318).

Having found that IFN-β could drive very limited IL-6 and sickness behaviour it was also necessary to establish the plausibility of the idea that acutely induced IFN-β was necessary for poly I:C-induced IL-6 (i.e. whether IFN-β was expressed significantly earlier than IL-6 after poly I:C stimulation *in vivo*). We examined the sequence of expression of IL-6 and IFN-β in the plasma and hippocampus of WT and IFNAR1^−/−^ mice 1 and 3 h post-challenge with saline or poly I:C (Fig. 4e–h). There was no detectable IL-6 or IFN-β in the plasma of saline-treated mice but 1 h post-poly I:C there was measureable plasma IL-6 and IFN-β and both these cytokines increased substantially in wild-type mice at 3 h. However, IL-6 concentration showed no further increase in the IFNAR1^−/−^ mice while IFN-β continued to rise at 3 h in both strains (Fig. 4e and f). Two-way ANOVA of plasma IL-6 showed a significant effect of strain (*F* = 13.41, df 1,25, *p* = 0.0012) and interaction between treatment and strain (*F* = 15.50, df 2,25, *p* = 0.0001). Bonferroni *post-hoc* analysis showed a significant difference between poly I:C and saline at 3 h in wild-type (*p* < 0.001) but not in IFNAR1^−/−^ mice. Two-way ANOVA for IFN-β showed equivalent responses to poly I:C in both strains (i.e. a significant effect of treatment (*F* = 44.39, df 2,25, *p* < 0.0001) but no significant effect of strain (*F* = 0.24, df 1,25, *p* = 0.6318), or interaction between these two factors (*F* = 0.39, df 2,25, *p* = 0.6818) indicating that IFNAR1 is necessary for the full poly I:C-induced plasma IL-6 response but not the plasma IFN-β response.

In the hippocampus IL-6 mRNA was not yet significantly induced in either strain at 1 h and was elevated only in the WT mice at 3 h post-poly I:C (Fig. 4g). Two-way ANOVA of IL-6 expression, with strain and treatment as factors, showed a significant effect of strain (*F* = 23.26, df 1,21, *p* < 0.0001) and treatment (*F* = 30.87, df 2,21, *p* = 0.0001) and an interaction between treatment and strain (*F* = 15.50, df 2,25, *p* < 0.0001). Bonferroni *post-hoc* analysis showed a significant difference in WT poly I:C-treated at 3 h when compared to controls (*p* < 0.001). The expression of IFN-β in the hippocampus was different to that seen in the plasma (Fig. 4h). There was detectable IFN-β expression present at 1 h in both strains, however IFN-β expression increased at 3 h post-poly I:C in WT mice but not in IFNAR1^−/−^ mice. There was a significant effect of strain (*F* = 5.10, df 1,21, *p* = 0.0347), treatment (*F* = 4.84, df 2,21, *p* = 0.0186), but no significant interaction (*F* = 2.25, df 2,21, *p* = 0.1303).

Thus, similar to plasma IL-6, the poly I:C-induced hippocampal IL-6 response is facilitated by IFNAR1. Moreover, in contrast to the plasma IFN-β response, poly I:C-induced hippocampal IFN-β is also mediated by IFNAR1 activity. Nonetheless, there was little evidence that acutely induced IFN-β might be responsible for the poly I:C-induced increase in IL-6 in WT mice. Rather the data suggest that IFNAR1 facilitates full IL-6 expression, perhaps through basal low level IFN-I activity.

### 3.4 STAT1 expression

It has been reported that STAT1 can contribute to the expression of IL-6 (Lee et al., 2006; Chaudhuri et al., 2008). Western blot analysis of STAT1 and phosphorylated STAT1 was performed in WT and IFNAR1^−/−^ mice to assess whether lower basal levels of STAT1 in IFNAR1^−/−^ mice might associate with the lack of IL-6 in the IFNAR1^−/−^ mice. STAT1 activity drives new transcription of STAT1 mRNA (Satoh and Tabunoki, 2013; Cheon and Stark, 2009) and we have thus also assessed stat1 mRNA as an additional measure of STAT1 activity when phospho STAT1 levels were too low to detect.

**Fig. 5:**
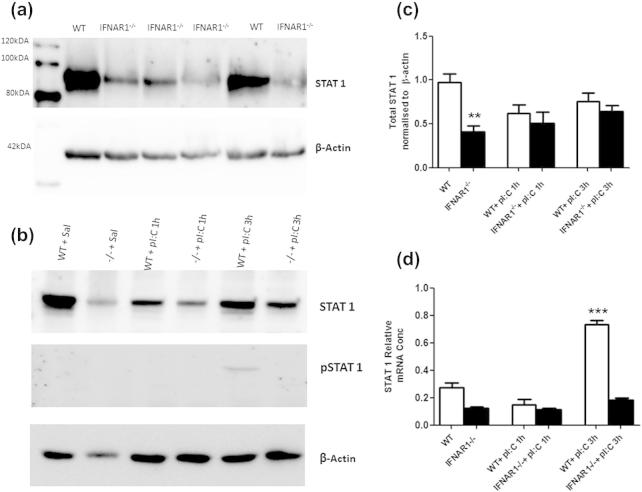
Expression of total and phosphorylated STAT1 in WT and IFNAR1^−/−^ mice. Western blot of basal STAT1 in WT and IFNAR1^−/−^ hippocampal homogenates (a). Western blot analysis of STAT1 and phosphorylated STAT1 in WT and IFNAR1^−/−^ mice post-poly I:C challenge at 1 and 3 h (b). Quantitative histogram of basal STAT1 in WT and IFNAR1^−/−^ mice and levels of STAT1 at 1 and 3 h post-poly I:C (c). Significant differences by Students *t* test are denoted by ^∗∗^*p* = 0.0015. Expression of stat1 mRNA (d). Data are expressed as mean ± SEM and analysed by two-way ANOVA with treatment and strain as factors; *n* = 5 for WT + saline, *n* = 4 for WT + pI:C 1 h, *n* = 5 for WT + pI:C 3 h and *n* = 5 for IFNAR1^−/−^ + saline, *n* = 4 for IFNAR1^−/−^ + pI:C 1 h, *n* = 5 for IFNAR1^−/−^ + pI:C 3 h. Significant main effect of treatment by Bonferroni *post-hoc* is denoted by ^∗∗∗^*p* < 0.001 *w.r.t.* saline-treated controls.

Western blotting showed that basal total STAT1 is higher in the WT than IFNAR1^−/−^ mice (Fig. 5a) and quantification by densitometry shows this difference to be significant (Student’s *t* test, *p* = 0.0015, Fig. 5c). Total and phospho-STAT1 were also assessed at 1 and 3 h post-poly I:C in WT and IFNAR1^−/−^ mice (Fig. 5b). Quantification (Fig. 5c) and analysis by two-way ANOVA with strain and treatment as factors demonstrate a significant effect of strain (*F* = 16.31, df 1,17, *p* = 0.0009) and a significant interaction (*F* = 7.61, df 1,17, *p* = 0.0134) but no effect of treatment (*F* = 0.01, df 1,17, *p* = 0.9389) indicating that poly I:C does not significantly alter total STAT1 levels within 3 h. STAT1 became phosphorylated (i.e. activated) at 3 h in all WT + poly I:C mice tested and was never detected in any other treatment/strain combination.

STAT1, which can translocate to neuronal nuclei upon phosphorylation (Gautron et al., 2003), has been shown to induce transcription of stat1 mRNA (Satoh and Tabunoki, 2013; Cheon and Stark, 2009) and therefore we also used hippocampal stat1 mRNA as a readout of STAT1 activity, when levels of phosphoSTAT1 were too low to detect (Fig. 5d). We found lower stat1 mRNA expression at baseline in IFNAR1^−/−^ mice with respect to WT mice (i.e. in saline-treated mice) and an acute induction of stat1 mRNA by 3 h. Predictably, this increase was visible only in WT polyI:C-treated animals. Two-way ANOVA showed a significant effect of strain (*F* = 149.93, df 1,15, *p* < 0.0001), treatment (*F* = 84.10, df 1,15, *p* < 0.0001) and a significant interaction (*F* = 49.98, df 1,15, *p* < 0.0001). Bonferroni *post-hoc* analysis revealed poly I:C treatment significantly increased STAT1 expression at 3 h, only in WT mice (*p* < 0.001 with respect to saline-treated controls), indicating that poly I:C-induced increases in hippocampal STAT1 expression are mediated by IFNAR1.

### 3.5 Genotype-dependent differences in basal and poly I:C-induced expression of inflammatory transcripts

**Fig. 6:**
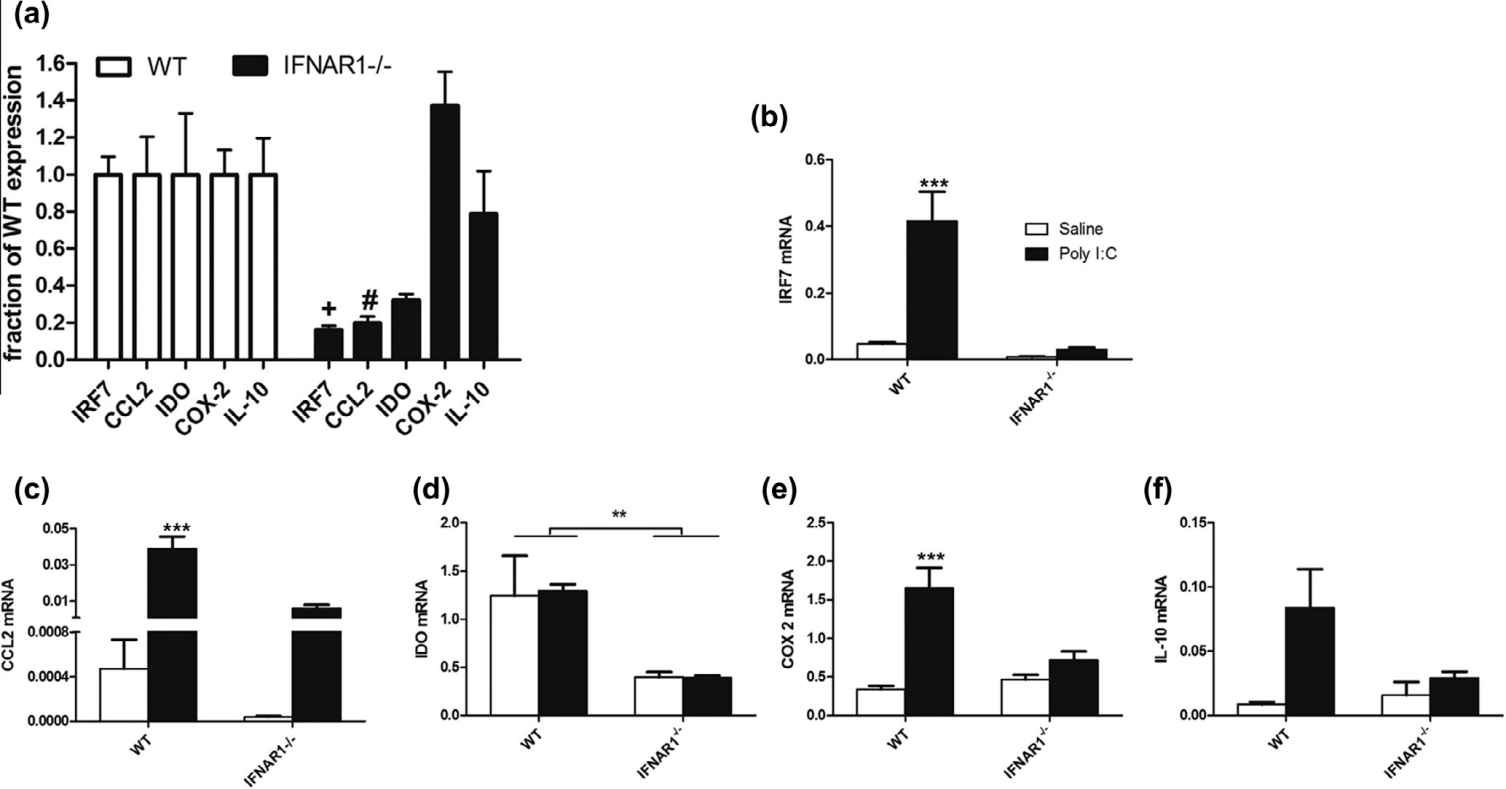
Differential basal and poly I:C-induced expression of inflammatory transcripts in WT and IFNAR1^−/−^ mice. (a) Basal expression of multiple inflammatory transcripts in IFNAR1^−/−^ mice are expressed as a fraction of WT expression levels and comparisons between WT and IFNAR1^−/−^ were made by Student’s *t* tests, with statistical significance denoted by ^+^*p* < 0.0001 and ^#^*p* = 0.0021. (b–f) Expression of the same transcripts 3 h post-challenge with saline or poly I:C (12 mg/kg i.p.): (b) IRF7, (c) CCL2, (d) IDO, (e) COX-2 and (f) IL-10. Data are expressed as mean ± SEM and analysed by two-way ANOVA with treatment and strain as factors, *n* = 5 for WT and *n* = 6 for IFNAR1^−/−^. Significant interactions between strain and treatment are denoted by ^*∗∗∗*^*p* < 0.001 and a significant main effect of strain is denoted by ^∗^*p* < 0.05 (d).

Given the basal differences in STAT1 expression, we predicted that there may be changes in basal levels of important STAT1-dependent inflammatory transcripts. A number of inflammatory transcripts with reported STAT1-dependent regulation were examined in the hippocampus of WT and IFNAR1^−/−^ mice (Fig. 6a). Interferon regulatory factor 7 (IRF7) is a transcription factor that is specifically induced by type I IFN action (via STAT1). CCL2-chemokine (C-C motif) ligand 2 (or monocyte chemoattractant protein-1) recruits monocytes, memory T cells, and dendritic cells to the sites of inflammation. Indoleamine-2,3-dioxygenase (IDO) is an enzyme that converts tryptophan to kynurenine. Cyclo-oxygenase 2 (COX-2) is a key prostaglandin biosynthetic enzyme and interleukin 10 (IL-10) is a key anti-inflammatory cytokine. Basal IRF7 and CCL2 are lower in IFNAR1^−/−^ than WT when expressed as a fraction of WT expression levels. Student’s *t* tests demonstrate significantly lower basal IRF7 (*p* < 0.0001) and CCL2 (*p* = 0.0021) in IFNAR1^−/−^ and lower levels of IDO that did not reach statistical significance (*p* = 0.1156). Neither COX 2 (*p* = 0.1454) nor IL-10 (*p* = 0.5340) were lower at basal levels.

Despite this lower basal level of CCL2, poly I:C still induces increased CCL2 expression in IFNAR1^−/−^ mice, but it remains lower than that observed in WT mice (Fig. 6c). Two-way ANOVA analysis revealed a significant effect of strain (*F* = 26.65, df 1,18, *p* < 0.0001), treatment (*F* = 47.56, df 1,18, *p* < 0.0001) and a significant interaction between strain and treatment (*F* = 25.28, df 1,18, *p* < 0.0001).

Transcription of the kynurenine biosynthetic enzyme indoleamine-2,3-dioxygenase (IDO), was analysed. Two-way ANOVA of IDO expression in the hippocampus (Fig. 6d) showed a significant effect of strain (*F* = 15.14, df 1,15, *p* = 0.0014) but no effect of treatment (*F* = 0.01, df 1,15, *p* = 0.9335) and no interaction (*F* = 0.02, df 1,15, *p* = 0.9023), indicating a decrease in basal hippocampal IDO expression in IFNAR1^−/−^ mice and no poly I:C-induced increase (at 3 h).

COX-2 and IL-10 expression (Fig. 6e and f) are similar in WT and IFNAR1^−/−^ at baseline but are less robustly induced 3 h post-poly I:C in IFNAR1^−/−^. Two way ANOVA analysis of COX-2 expression also showed a significant effect of strain (*F* = 7.95, df 1,18, *p* = 0.0113), treatment (*F* = 29.70, df 1,18, *p* < 0.0001) and a significant interaction (*F* = 13.75, df 1,18, *p* = 0.0016). Two way ANOVA analysis of IL-10 expression showed a significant effect of treatment (*F* = 7.04, df 1,15, *p* = 0.0181) but not of strain (*F* = 2.06, df 1,15, *p* = 0.1718) and no interaction (*F* = 3.40, df 1,15, *p* = 0.0850) indicating that the poly I:C-induced increase in hippocampal COX-2 and IL-10 expression is attenuated in IFNAR1^−/−^ mice.

Therefore, while the expression of IRF7, CCL2, IDO, COX-2 and IL-10 are all significantly different in poly I:C-treated IFNAR animals with respect to wild-types, lower basal expression of IRF7, CCL2 and IDO certainly contributes to this effect.

### 3.6 Kynurenine and tryptophan

**Fig. 7:**
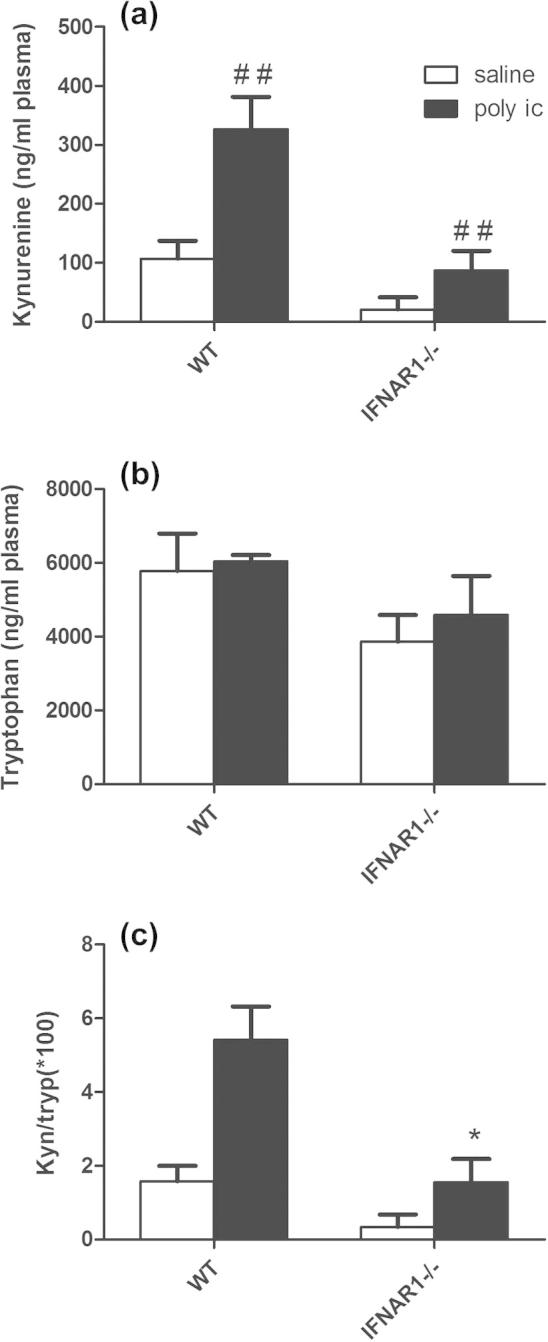
Levels of kynurenine pathway metabolites in WT and IFNAR1^−/−^ mice. Levels of kynurenine (a), tryptophan (b) and the ratio of kynurenine to tryptophan (c) in the plasma of WT and IFNAR1^−/−^ mice were assessed by HPLC 24 h post-poly I:C (12 mg/kg). Data are expressed as mean ± SEM and analysed by two-way ANOVA with treatment and strain as factors, *n* = 5 for WT and *n* = 6 for IFNAR1^−/−^. Significant main effect of treatment is denoted by ^*##*^*p* < 0.01 and significant interaction between strain and treatment is denoted by ^*∗*^*p* < 0.05.

We were unable to detect reliable levels of the IDO product kynurenine by HPLC in either the hippocampus or frontal cortex of these animals but levels of plasma kynurenine were clearly higher in WT animals than in IFNAR1^−/−^ mice (Fig. 7a). Two-way ANOVA shows a significant effect of strain (*F* = 19.68, df 1,17, *p* = 0.0004), treatment (*F* = 15.09, df 1,17, *p* = 0.0012) but no significant interaction (*F* = 4.39, df 1,17, *p* = 0.0515). Poly I:C induced a robust elevation of kynurenine and of the kynurenine:tryptophan ratio 24 h post-poly I:C (Fig. 7a and c) and this was significantly attenuated in IFNAR1^−/−^ mice. Two-way ANOVA of the kynurenine:tryptophan ratio with strain and treatment as factors shows a significant effect of strain (*F* = 16.94, df 1,17, *p* = 0.0007), treatment (*F* = 16.62 df 1,17, *p* = 0.0008) and a significant interaction (*F* = 4.49, df 1,17, *p* = 0.0491).

Thus, although a number of inflammatory markers show restricted expression upon challenge with poly I:C, we cannot attribute these differences solely to a lack of responsiveness to acutely induced IFN-I: these animals express lower levels of these markers at baseline, and although poly I:C-induced elevations remain possible, robust elevation is facilitated by the expression of IFNAR1.

### 3.7 IL-6 supplementation of poly I:C challenges

**Fig. 8:**
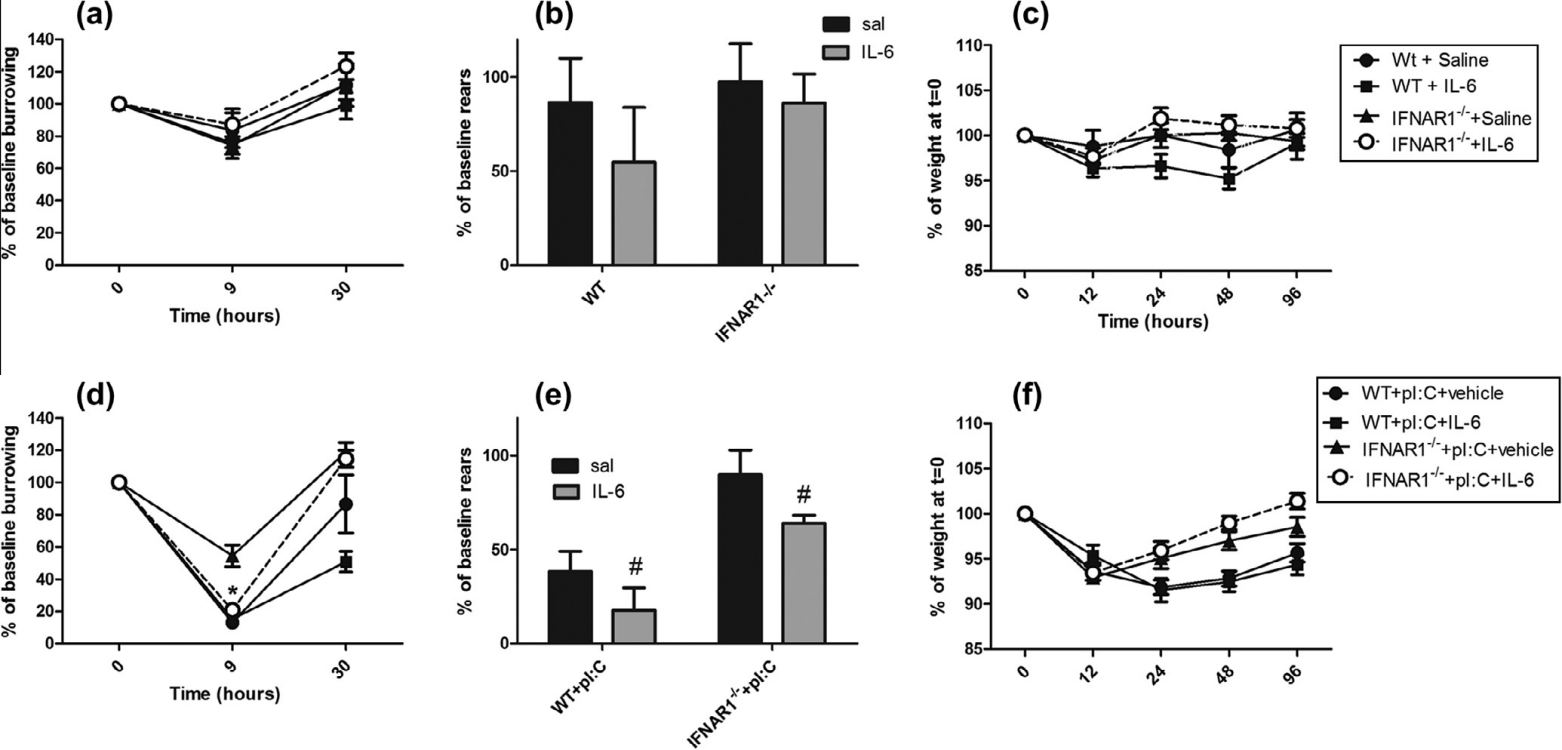
Sickness behaviour post-IL-6 and post-poly I:C ± IL-6 challenges. (a–c) The sickness response of C57BL/6 and IFNAR1^−/−^ mice to saline or interleukin-6 (50 μg/kg i.p.), as assessed by burrowing (a), rearing activity in the open field (b) and % body weight loss (c). Data are expressed as mean ± SEM, *n* = 8 for WT + saline, *n* = 7 for WT + IL-6, *n* = 10 for IFNAR + saline, *n* = 10 for IFNAR + IL-6. (d–f) The sickness response of C57BL/6 and IFNAR1^−/−^ mice to poly I:C + vehicle (12 mg/kg i.p.) versus poly I:C + IL-6 (50 μg/kg i.p.) challenges on burrowing (d), rearing (e) and body weight (f). Data are expressed as mean ± SEM, *n* = 11 for WT + pI:C and IFNAR + pI:C, *n* = 12 for IFNAR + pI:C + IL-6 and *n* = 5 for WT + pI:C + IL-6. Rearing activity at 3 h was analysed by two-way ANOVA (b, e) with strain and treatment as factors. Burrowing at 9 h (a, d) and body weight change (c, f) were analysed by three-way ANOVA with strain, treatment and time as factors. * denotes significant difference between IFNAR + pI:C + vehicle and IFNAR + pI:C + IL-6 at 9 h after a significant two-way ANOVA at that time (*p* < 0.05). # denotes significant main effect of IL-6 treatment by two-way ANOVA (e).

Returning to the original observations in this study: since IL-6 responses to poly I:C challenge are very significantly deficient, we hypothesised that the sickness behaviour response to poly I:C could be reconstituted by co-injecting exogenous IL-6 with poly I:C in IFNAR1^−/−^ animals. To facilitate those experiments we first determined the effects of IL-6 alone in wild type and IFNAR1^−/−^ mice (Fig. 8a–c). For these experiments we omitted measurements of core body temperature, since the effects of poly I:C were somewhat modest and variable with rectal probe measurements, and replaced this with burrowing which is an extremely sensitive measure of suppression of species typical behaviours (Deacon, 2006).

Burrowing activity was measured at 9 and 30 h, open field activity at 3 h and weight loss at 12, 24 and 48 h post-i.p. challenge with IL-6 (50 μg/kg) or saline. There were no statistically significant effects of IL-6 treatment or strain on any of these parameters using three way ANOVA (*F* ⩽ 0.99, df 1,31, *p* ⩾ 0.3276).

Poly I:C induced a decrease in burrowing in all groups but, as before, this decrease was attenuated in IFNAR1^−/−^ mice. However, when IL-6 was co-administered with poly I:C, burrowing in IFNAR1^−/−^ mice was decreased to levels equivalent to those in WT + poly I:C animals (Fig. 8d). That is, IL-6 could reconstitute in IFNAR1^−/−^ mice the full deficit observed after poly I:C treatment in WT animals. We made the *a priori* prediction that burrowing would be maximally affected at 9 h and a two-way ANOVA analysis of the four poly I:C-treated groups at this time showed a main effect of strain (*F* = 18.49, df 1,31, *p* = 0.0002), of IL-6 (*F* = 8.29, df 1,31, *p* = 0.0072) and an interaction between these factors (*F* = 10.74, df 1,31, *p* = 0.0026). Bonferroni *post-hoc* analysis of these data showed that when IL-6 was added simultaneously with poly I:C, burrowing in the IFNAR1^−/−^ mice was no longer significantly different to poly I:C in the wild-type mice (*p* >> 0.05), while those challenged with poly I:C + vehicle burrowed significantly more (*p* < 0.001 with respect to poly I:C + IL-6). Thus, the poly I:C-induced decrease in burrowing, which is absent in the IFNAR1^−/−^, is dependent on IL-6 levels.

We previously observed that poly I:C had less effect on rearing activity at 3 h in IFNAR1^−/−^ mice (Fig. 2b). Therefore, we examined the ability of poly I:C + IL-6 to block rearing activity in IFNAR1^−/−^ mice, compared to poly I:C in WT mice (Fig. 8e). Examining the effect of strain and co-administration of IL-6 at 3 h, two-way ANOVA revealed a significant effect of IL-6 co-treatment (*F* = 4.43, df 1,35, *p* = 0.0425) and of strain (*F* = 19.57, df 1,35, *p* < 0.0001) but no significant interaction (*F* = 0.05, df 1,35, *p* = 0.8213). Thus IL-6 exacerbates poly I:C-induced suppression of rears, but does so equally in WT and IFNAR1^−/−^ animals. In other words, both IL-6 and IFNAR1 influence poly I:C-induced decrease in rears but these effects are independent of each other.

Poly I:C induced a marked weight loss in both strains as previously observed, but this recovered more quickly in IFNAR1^−/−^ mice (Fig. 2c). We addressed the possibility that the addition of IL-6 with poly I:C might reconstitute the effect of poly I:C in WT animals. However the addition of IL-6 did not slow the recovery of weight in poly I:C-treated IFNAR1^−/−^ mice (Fig. 8f). A three-way ANOVA of these data, using strain and IL-6 as between subjects factors and time as the within subjects factor showed a main effect of strain (*F* = 21.71, df 1,38, *p* < 0.0001) but no effect of IL-6 (*F* = 1.18, df 1,38, *p* = 0.283) and no interaction of strain and IL-6 (*F* = 1.37, df 1,38, *p* = 0.2496). Therefore the impact of type I interferon on weight loss during poly I:C-induced sickness behaviour does not appear to be mediated or modulated by IL-6.

Collectively these data show that IFNAR1 contributes to the sickness behaviour responses in both IL-6-dependent and independent mechanisms.

## 4 Discussion

We demonstrate robust CNS expression of IFN-β, but not IFN-α, after systemic poly I:C and have demonstrated that several CNS behavioural and metabolic effects of poly I:C are attenuated in IFNAR1^−/−^ mice. The IL-6 response to poly I:C is profoundly diminished in IFNAR1^−/−^ mice and this is associated with a lack of basal IFN-I and STAT1 expression. Addition of IL-6 is sufficient to reconstitute full expression of some measures of altered CNS function such as burrowing, but not anorexia. These data demonstrate important interdependent and independent roles of IFNAR1 and IL-6 signalling in the context of sickness and CNS behavioural responses to systemic inflammation induced by viral mimetics.

### 4.1 IFNAR1 deficiency influences the sickness behaviour response

Sickness behaviour is different in IFNAR1^−/−^ mice. The subjective observation that IFNAR1^−/−^ mice ‘appeared’ less sick, based on less hunched posture and ruffled fur was borne out in objective measures of the sickness response. We examined rearing behaviour in the open field, an exploratory behaviour that was significantly reduced by poly I:C but less so in IFNAR1^−/−^. There was also less marked hypothermia in IFNAR1 deficient mice. The reduction of poly I:C-induced hypothermia in IFNAR1^−/−^ is consistent with data showing protection in IFNAR1^−/−^ against the robust and progressive hypothermia a number of days after inoculation with influenza virus in wild type mice (Traynor et al., 2007). Prior studies also indicate a hypothermic effect of IFN-α (Sammut et al., 2001) and a hyperthermic effect of IFN-β during the light phase (Billiau et al., 1980; Dinarello et al., 1984; Ohdo et al., 1997). Burrowing is a rewarding behaviour in mice (Deacon, 2006). Our studies indicate that poly I:C induced an effect on burrowing, which may be described as anhedonic, although we cannot rule out the possibility that this simply reflects reduced activity in a more general sense. Nevertheless, the poly I:C-induced burrowing deficit was largely abolished in IFNAR1^−/−^ and this effect was mediated via the deficient IL-6 response in IFNAR1^−/−^ mice, which differs somewhat from the IL-6 dependence of the locomotor activity deficits. Some recent data suggest that decreased wheel-running in poly I:C-treated animals is not prevented by systemic administration of an anti-IFN-β antibody (Matsumoto et al., 2008). The direct administration of IFN-β, in the current study, had statistically significant, but very mild effects on open field activity and burrowing (Fig. 4). These mild effects may be explained by the rapid clearance of injected cytokines (Abraham et al., 2010) but may also reflect the requirement for IL-6 to mediate or contribute additively to IFNAR effects (Fig. 8).

Poly I:C is known to produce robust anorexia (Cunningham et al., 2007). A clear poly I:C-induced anorexic effect was also experienced in IFNAR1^−/−^ mice in the current study but its duration was much shorter than in wild-type mice, indicating a significant contribution of IFN-I to anorexia. Poly I:C-treated animals remained capable of rearing and climbing and, in previous studies, maintained muscle strength and motor coordination (Field et al., 2010) and it is therefore highly likely that weight loss reflected decreased drive to eat rather than inability to obtain food. This effect was not altered by IL-6 addition, consistent with a direct role for IFN-I in mediating this effect, in agreement with early reports of effects of IFN-α on food intake (Segall and Crnic, 1990). Although most animal studies of sickness-inducing IFN-I effect have focussed on IFN-α, we only observed increased IFN-β in the current study and there are human data to indicate that IFN-β produces significant fatigue and anorexia (Rinehart et al., 1986; Borden et al., 2011). However, weight loss occurred rapidly in IFNAR1^−/−^ mice and therefore must be independent of IFN-I, perhaps occurring via TNF-α, which is known to contribute to cachexia and whose expression is increased by poly I:C and not significantly diminished in IFNAR1^−/−^ mice (Fig. 3).

In describing effects of IFNAR on sickness, it is important to recognise that type I IFN is essential in combating viral infection and the absence of the type I IFN response during active infection would be predicted to, and indeed has been shown to, lead to increased sickness as the animal fails to control the infection (Kugel et al., 2009).

### 4.2 Impaired IL-6 response to poly I:C in IFNAR1^−/−^ mice

In order to delineate why these behavioural and metabolic responses in the CNS are different in animals unresponsive to type I IFN, we first assessed the cytokine profile in blood and brain, revealing some striking differences. The IL-6 response to poly I:C was profoundly diminished in IFNAR1^−/−^ mice indicating that robust IL-6 expression upon poly I:C challenge is type I IFN-dependent. The simplest explanation of this effect would be that, in normal animals, poly I:C induces IFN-β, which in turn induces IL-6. However this is not intuitive since NFκB is a key regulator of IL-6 and is induced rapidly after LPS, TNF-α or double stranded RNA stimulation (Libermann and Baltimore, 1990) and indeed this explanation does not fit with the data in the current study. Firstly, intraperitoneal challenge with IFN-β produced very limited IL-6 expression in blood and brain, consistent with the limited evidence for IFN-α/β directly inducing IL-6 (Ito et al., 1996). Secondly, plasma analysis at 1 and 3 h post-poly I:C (Fig. 4) demonstrated that IFN-β does not precede IL-6 expression. However, IL-6 continues to increase in WT animals at 3 h but this further increase in IL-6 is absent in IFNAR1-deficient mice. These data indicate that acute IFN-β expression is not the signal that triggers IL-6 expression. Rather, we propose that basal IFN-I expression is necessary to facilitate the robust expression of IL-6 upon exposure to poly I:C. There is now ample evidence that low level actions of type I interferons are essential to enhance cellular responses to subsequent stimuli (Taniguchi and Takaoka, 2001) and these include optimisation of both type I interferon responses but also responses to other cytokines such as IFN-γ and IL-6 (Muller et al., 1994; Mitani et al., 2001). It is also known that IFN-γ-induced factors can synergise with TLR agonists to produce optimal levels of TLR target genes and key among these factors is STAT1, which synergises with NFκB to maximise inflammatory mediator output (Schroder et al., 2006). STAT1 activity is also a major signalling mechanism in type I interferon actions and it has been described that cells that are IFNAR1^−/−^ provide less docking sites for STAT1 and STAT3, thus impairing STAT1/3 and therefore also IL-6 and IFN-γ signalling (Mitani et al., 2001). However IFNAR1^−/−^ animals have suppressed levels of total STAT1 with respect to normal animals and this lower STAT1 has been shown to result in lower expression of STAT1-dependent genes *stat1*, *irf1* and *junB* (Gough et al., 2010), providing a simpler explanation of less efficient expression of STAT1-dependent genes in IFNAR1^−/−^ mice. Our data replicate, in the brain, this lower STAT1 expression level, decreased STAT1 phosphorylation post-poly I:C and the decreased transcription of the dependent gene *stat1* (Fig. 5). This suggests that basal STAT1 has a role in facilitating poly I:C-induced IL-6 in the CNS, probably in co-operation with NFκB, which is also known to influence IL-6 expression after double stranded RNA stimulation (Libermann and Baltimore, 1990). JAK-STAT1 signalling has been shown to be sufficient for IL-6 expression in macrophages treated with phosphatidic acid (Lee et al., 2006) and IRF1 and NFκB have also been shown to co-operate, at low levels of expression, at the IL-6 promoter (Faggioli et al., 1997). In HIV infection of cultured cells, the STAT1 inhibitor fludarabine significantly decreased IL-6 expression (Chaudhuri et al., 2008) indicating the importance of STAT1 signalling for IL-6 expression during viral infection. The regulation of poly I:C-induced IL-6 by IFNAR1 via STAT1 still requires further elucidation.

### 4.3 Role of basal IFNAR1 signalling in inflammatory regulation in the brain

While IL-6 showed a very much reduced expression in response to poly I:C, it is not the only transcript significantly lower in IFNAR1^−/−^ than in wild types. Other inflammatory transcripts including IRF7, CCL2 and IDO were significantly less robustly induced in IFNAR1^−/−^ mice (Fig. 6). However there were already significant differences between strains at the basal level. These data suggest that basal activity of IFNAR1 (likely activated by IFN-β, but we cannot rule out low levels of IFN-α) influences the basal expression of CCL2, IDO and perhaps many other inflammatory transcripts. These transcripts appear to remain responsive to poly I:C stimulation, but never to the same levels as in WT mice, and the origins of this lower expression may lie in the facilitating effects of basal actions of IFN-I, not least via basal expression of STAT1. Like IL-6, CCL2 transcription is also regulated by NFκB, but there are studies showing that STAT1 co-regulates its induction (Valente et al., 1998; Sikorski et al., 2014) and the current study demonstrates that CCL2 transcripts are lower both at baseline and post-poly I:C, congruent with the total STAT1 levels. IDO expression is also known to be induced by IFN-I in a STAT1-dependent manner (Du et al., 2000).

We also found that circulating tryptophan and kynurenine levels were lower both basally and after poly I:C induction in IFNAR1^−/−^ mice with respect to WT controls. IDO expression was also reduced in IFNAR1^−/−^ mice. These data suggest that IFN-I is important in regulating the production of metabolites on this pathway. Since KYN can cross the blood brain barrier (Fukui et al., 1991) elevated plasma levels of KYN may also influence brain concentrations and indeed blocking the peripheral production of KYN, via inhibition of liver tryptophan-2,3-dioxygenase, produces anti-depressant effects in a repeated restraint stress model of depression (Gibney et al., 2014). It is also possible, therefore, that basal IFN-I actions influence depressive states. We did not observe statistically significant behavioural differences between the strains at baseline but more comprehensive analysis of basal differences is warranted on the basis of the observed differential basal activation/expression of inflammatory genes/pathways.

### 4.4 Does diminished IL-6 explain the altered sickness behaviour response?

There is evidence that IL-6 contributes to sickness behaviour and thermoregulatory responses. Centrally injected IL-6 increased body temperature and suppressed locomotor activity and food intake in rats (Schobitz et al., 1995), although in other studies effects of centrally administered IL-6 on locomotor activity were not observed even at doses significantly higher than those producing febrile and hypothalamic pituitary adrenal axis responses (Lenczowski et al., 1999). Nonetheless, when co-administered with IL-1β in the same study, IL-6 increases the severity of suppression of social interaction and immobility. Consistent with a contributory rather than direct causative role, deletion of the IL-6 gene in mice attenuates sensitivity to the depressing effects of LPS and IL-1 on social exploration and body weight (Bluthe et al., 2000) and using soluble gp130 (sgp130) to block IL-6 trans-signalling also reduces LPS-induced suppression of locomotor activity (Burton et al., 2013). We demonstrate altered thermoregulatory and sickness behaviour responses to poly I:C in IFNAR1^−/−^. The effects of poly I:C on burrowing appear to be mediated by IL-6, consistent with reported anhedonic effects of IL-6. However, the effects on open field rears are independent of IL-6, although IL-6 itself further decreases rearing. IDO has also been implicated in anhedonia emerging from sickness episodes: blocking IDO activity can limit LPS-induced depressive-like behaviour (O’Connor et al., 2009) and could have a role in the IFNAR1^−/−^ and IL-6-dependent burrowing deficits observed here.

Another possible mediator of the muted response of IFNAR1^−/−^ mice to poly I:C might be COX-2, which is crucial in a number of aspects of the sickness behaviour responses including thermoregulatory responses (Saper et al., 2012). COX-2 is robustly induced by systemic poly I:C (Cunningham et al., 2007) and its mRNA induction was muted in IFNAR1^−/−^ in the current study (Fig. 6). The contribution of COX-2 to poly I:C-induced sickness behaviour requires further study, although preliminary data suggests robust vascular COX-2 expression remains in IFNAR1^−/−^ animals.

## 5 Conclusion

This study makes it clear that the induction of type I interferons during peripheral innate immune activation, mimicking viral infection, has effects on CNS-mediated behavioural and metabolic changes by IL-6-dependent and independent mechanisms. Basal activity of the type I IFN receptor affects basal STAT1 levels which appear to alter basal expression of inflammatory transcripts in the CNS and facilitates the brain’s inflammatory response to systemic challenge with double stranded RNA. Since there is now evidence that systemic poly I:C and type I IFN responses can affect progression of neurodegenerative pathology (Field et al., 2010; Wang et al., 2004; Khorooshi et al., 2013) and cognitive decline in aging (Baruch et al., 2014), and STAT1 is an important regulator of microglial phenotype (Lawrence and Natoli, 2011) it is also pertinent to investigate the microglial phenotype of IFNAR1^−/−^ mice in models of microglial activation and chronic neurodegeneration.
