# Supplementary material for: Interdependent and independent roles of type I interferons and IL-6 in innate immune, neuroinflammatory and sickness behaviour responses to systemic poly I:C
Source: Brain Behav Immun. 2015 Aug;48:274–86. doi: 10.1016/j.bbi.2015.04.009 (PMC4521083; doi:10.1016/j.bbi.2015.04.009)
Supplement: Supplementary Figs. S1 and S2 [file mmc1.doc]

**Supplementary material.**


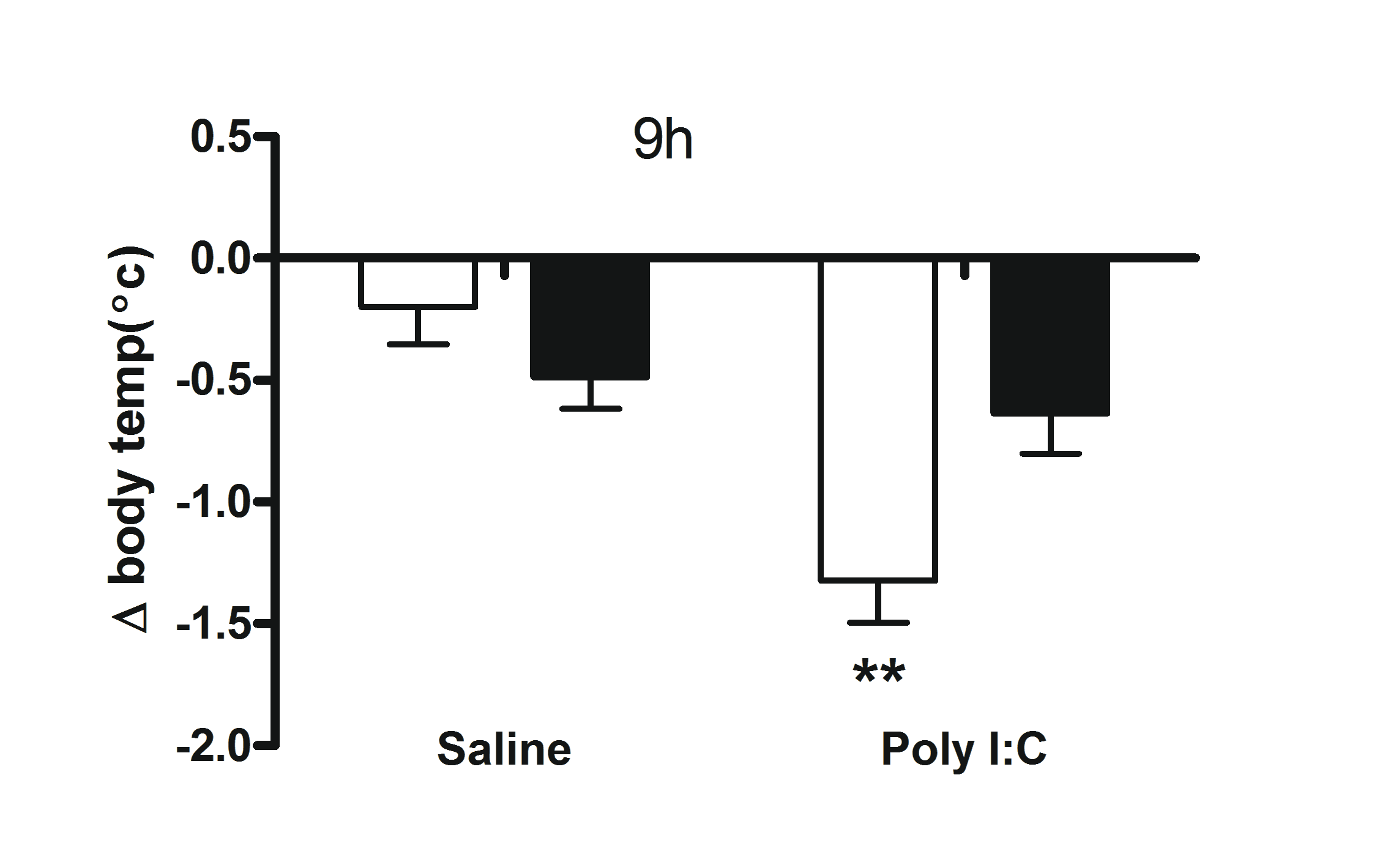


**Figure S1. Hypothermia at 9 hours post-poly I:C (12 mg/kg).**

Core-body temperature was measured using a rectal probe at 9 hours post-poly I:C at 12 mg/kg. As previously described (Cunningham et al., 2007), poly I:C produced robust hypothermia in wild-type animals (**white bars**) that was less marked in IFNAR1^-/-^ mice (**black bars**). Two-way ANOVA showed a significant effect of treatment (F=13.91, df 1,40, p=0.0006), no effect of strain (F=1.37, df 1,40, p=0.2489) but a significant interaction between treatment and strain (F=8.22, df 1,40, p=0.0066) indicating the more limited hypothermic response in IFNAR1^-/-^ mice.

**
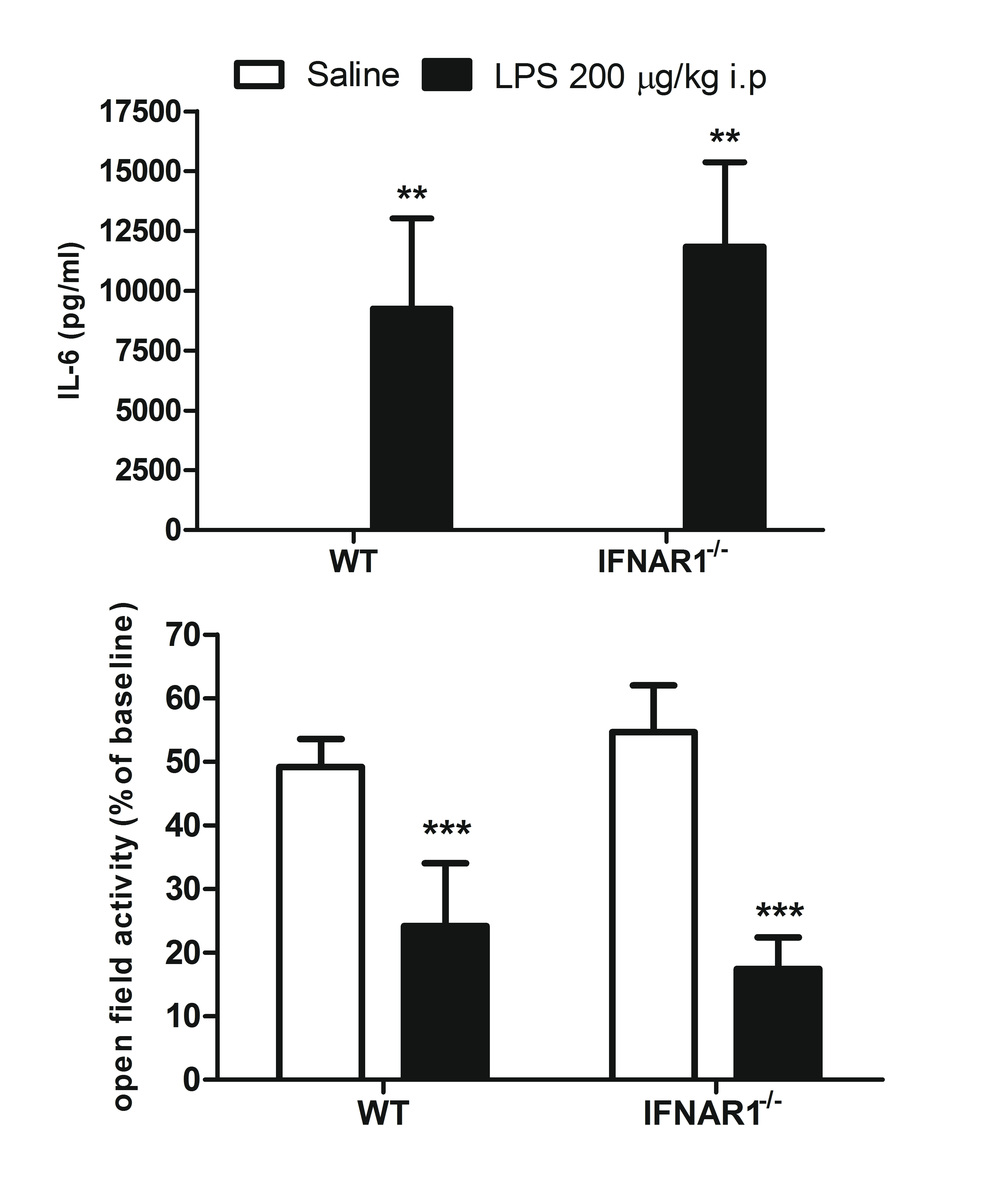
**

**Figure S2. LPS-induced sickness behaviour and IL-6 expression in wild-type and IFNAR1^-/-^ mice.** Animals were challenged intraperitoneally with LPS (200 µg/kg) and assessed on open field activity at 170 minutes before collection of blood and ELISA assay of plasma IL-6 (3 hours). LPS significantly increased IL-6 (top panel, ** denotes main effect of LPS, p<0.01) but this effect was not different between WT and IFNAR1^-/-^ mice. Similarly, open field activity was reduced to below 30% in both strains (*** denotes main effect of LPS, p<0.001) but this was significantly different between strains. Thus LPS produces equal IL-6 and locomotor activity suppression in wild-type and IFNAR1^-/-^ mice.
